# Health-related quality of life in food hypersensitive schoolchildren and their families: parents' perceptions

**DOI:** 10.1186/1477-7525-4-48

**Published:** 2006-08-10

**Authors:** Birgitta Marklund, Staffan Ahlstedt, Gun Nordström

**Affiliations:** 1 Centre for Allergy Research, Karolinska Institutet, S-171 77 Solna, Sweden; 2 Department of Neurobiology, Care Sciences and Society, Division of Nursing, 23300, Karolinska Institutet, S-141 83 Huddinge, Sweden; 3 National Institute of Environmental Medicine, Karolinska Institutet, S-171 77 Solna, Sweden; 4 Division of Health and Caring Sciences, Karlstad Universitet, S-651 88 Karlstad, Sweden

## Abstract

**Background:**

About 20% of schoolchildren and adolescents in Sweden suffer from perceived food hypersensitivity (e.g. allergy or intolerance). Our knowledge of how child food hypersensitivity affects parents HRQL and what aspects of the hypersensitivity condition relate to HRQL deterioration in the family is limited. Thus the aim of this study was to investigate the parent-reported HRQL in families with a schoolchild considered to be food hypersensitive. The allergy-associated parameters we operated with were number of offending food items, adverse food reactions, additional hypersensitivity, allergic diseases and additional family members with food hypersensitivity. These parameters, along with age and gender were assessed in relation to child, parent and family HRQL.

**Methods:**

In May 2004, a postal questionnaire was distributed to parents of 220 schoolchildren with parent-reported food hypersensitivity (response rate 74%). Two questionnaires were used: CHQ-PF28 and a study-specific questionnaire including questions on allergy-associated parameters. In order to find factors that predict impact on HRQL, stepwise multiple linear regression analyses were carried out.

**Results:**

An important predictor of low HRQL was allergic disease (i.e. asthma, eczema, rhino conjunctivitis) in addition to food hypersensitivity. The higher the number of allergic diseases, the lower the physical HRQL for the child, the lower the parental HRQL and the more disruption in family activities. Male gender predicted lower physical HRQL than female gender. If the child had sibling(s) with food hypersensitivity this predicted lower psychosocial HRQL for the child and lower parental HRQL. Food-induced gastro-intestinal symptoms predicted lower parental HRQL while food-induced breathing difficulties predicted higher psychosocial HRQL for the child and enhanced HRQL with regards to the family's ability to get along.

**Conclusion:**

The variance in the child's physical HRQL was to a considerable extent explained by the presence of allergic disease. However, food hypersensitivity by itself was associated with deterioration of child's psychosocial HRQL, regardless of additional allergic disease. The results suggest that it is rather the risk of food reactions and measures to avoid them that are associated with lower HRQL than the clinical reactivity induced by food intake. Therefore, food hypersensitivity must be considered to have a strong psychosocial impact.

## Background

About 20% of schoolchildren and adolescents in Sweden suffer from perceived hypersensitivity (e.g. allergy or intolerance) to defined food items [1,2]. The way such allergy-associated condition is manifested varies extensively depending on the specific foods and the type of reactions and symptoms that the child has. Adverse reactions to food can result in either immediate or delayed problems depending on the mechanism underlying the reaction. Food reactions can range from mild (e.g. mild itching or redness around the mouth), unpleasant and scary (e.g. intense stomach pains or asthma) to systemic reactions severe enough to be fatal (i.e. anaphylaxis) [3]. As there is no satisfying treatment for most kinds of food hypersensitivity individuals have to manage their problems by themselves and, if possible, try to avoid the offending food.

It is not uncommon that individuals with food hypersensitivity have other hypersensitivities or allergy-associated problems as well [4-6]. Food allergy is associated with other manifestations of atopy, such as urticaria, asthma, atopic eczema and hay fever [7]. Our previous study showed that 78% of food hypersensitive Swedish schoolchildren may experience such symptoms that can be related to asthma, eczema and/or rhino conjunctivitis [2]. A number of studies describe deterioration of the health-related quality of life (HRQL) in children and adults with the allergic diseases asthma [8-11], eczema [12-15] and rhinitis [16-19]. However, physical and organ-related measures and tests do not always correlate with HRQL scores [20-22]. Thus there is no absolute coherence between the biomedical severity of the allergy-associated condition and quality of life.

In recent years there have been a number of studies that show that food hypersensitivity impair the HRQL of individuals and even their families [23-26]. Our recent school survey [2] showed that those young individuals who, in addition to their food related problems suffered from other allergy-associated conditions, reported a greater impact on the physical life quality dimension than those without such chronic problems. The psychosocial impact of food hypersensitivity was however reported equally low regardless of other allergy-associated conditions [2]. Avery et al [24] have shown that children with peanut allergy have a poorer HRQL, apparently related to anxiety, compared with children with diabetes. Studies from Sicherer et al [25] and Primeau et al [26] have demonstrated a psychosocial impact of food allergy on quality of life on both the child and its family. It has also been shown that the parents of food allergic children experience more distress and worry, and also more interruptions and limitations in usual family activities, compared with a US population sample [25]. Furthermore, the parents of peanut-allergic children report significantly more disruption in the child's daily activities and in their familial-social interactions than parents of children with rheumatological diseases [26].

It is first and foremost the children's parents who are responsible for not exposing their children to potentially dangerous dietary products, and food hypersensitivity in a child often leads to the whole family having to adapt to new food and eating practices. Yet, little is known of how child food hypersensitivity affects parents' HRQL and what aspects of the hypersensitivity condition relate to HRQL deterioration in the family. Thus the aim of the study was to investigate the parent-reported HRQL in families with a schoolchild considered to be food hypersensitive. The following research questions were addressed:

• How do parents of a food hypersensitive child perceive the child's, their own and the family's HRQL?

• To what extent do allergy-associated parameters, age and gender relate to child, parent and family HRQL?

Allergy-associated parameters in this study include number of offending food items, adverse food reactions, additional hypersensitivities, allergic diseases and additional family members with food hypersensitivity.

## Methods

### Study population and procedure

In May 2004, a postal questionnaire (see below) was distributed to parents of 220 schoolchildren with parent-reported food hypersensitivity. The schoolchildren were pupils at the nine-year compulsory school and at the upper secondary school in a municipality in the south of Stockholm, Sweden. In 2002 this group of parents had participated in a previous study on allergy-associated conditions and health-care contacts of children with exclusion diets at school [27]. The previous study included parents of 230 schoolchildren, of whom 10 children were excluded from the present study as they had left school. The sample procedure for the previous study, and thus for the present study as well, has been described in detail elsewhere [27].

In the present study, twenty of the 220 questionnaires came in return with the information that the addresses were inaccurate. After two weeks a reminder letter was sent out to all the parents of the remaining 200 schoolchildren. A total of 147 questionnaires were sent back (74% answer rate). Of the 147 questionnaires eight parents notified that their child was no longer hypersensitive to food, and another five questionnaires were omitted for missing data. Thus 134 questionnaires provide the information that the following results are based on.

### Questionnaires

Two questionnaires were used: the Child Health Questionnaire – Parent-completed Form 28 (CHQ-PF28) [28] and a study-specific questionnaire.

### Child Health Questionnaire – Parent-completed Form 28

The generic instrument Child Health Questionnaire – Parent-completed Form 28 (CHQ-PF28) was used to measure HRQL in a child and its family. The CHQ-PF28 consists of 28 items, which refer to 13 health scales: nine scales measure the well-being of the child, two scales measure the impact of child's health on parents' HRQL and two scales measure the impact on the family. In order to make it possible to interpret the results across the scales, the raw scale scores are transformed to standardized 0 to 100 scores by using defined algorithms, with lower scores signifying lower HRQL. Ten of the 13 scales are summed up into two comprehensive health scales representing the child's physical and psychosocial well-being (see Table 1). These summary measures are standardized so that all scores above and below 50 are above and below the average, respectively, of a general U.S. child population [28]. One scale concerning the change of the child's health during the last 12 months is not used in the present study.

**Table 1:** Summary of contents and Cronbach's alpha of the CHQ-PF28 health scales [28]

**Scales**	**Abbr**.	**Summary of content**	**No. of items**	**Cronbach's alpha**^**1**^
Physical Functioning	PF	Child's limitations in performing physical activities, including self-care, due to health.	3	0.85
Role/social limitations-Emotional-Behavioural	REB	Child's limitations in school work or activities with friends as a result of emotional and/or behavioural problems.	1	
Role/social limitations-Physical	RP	Child's limitations in schoolwork or activities with friends as a result of physical health.	1	
Bodily Pain	BP	Child's degree and frequency of bodily pain.	1	
General Behaviour	BE	Child's frequency of behavioural problems, e.g. exhibits aggressive, immature, delinquent behaviour.	4	0.74
Mental Health	MH	Child's frequency of positive and negative feelings, e.g. anxiety, depression, happiness and peacefulness.	3	0.68
Self Esteem	SE	Child's satisfaction with abilities, looks, family/peer relationships and life overall.	3	0.78
General Health	GH	Child's past, future and current health.	4	0.65
Parental impact-Emotional	PE	Parent's experience of emotional worry/concern as a result of child's physical and/or psychosocial health.	2	0.65
Parental impact-Time	PT	Parent's experience of limitations in time available for personal needs due to child's physical and/or psychosocial health.	2	0.75
Family Activities	FA	Family's frequency of disruption in family activities due to child's health.	2	0.74
Family-Cohesion	FC	Family's ability to get along.	1	
Change in Health^2^	CH	Child's health as compared to a year ago.	1	

Physical Summary measure	PhS	Summary measure for the physical dimension of the CHQ.		0.80
Psychosocial Summary measure	PsS	Summary measure for the psychosocial dimension of the CHQ.		0.84

^1^Cronbach's alpha show the internal consistency in the present study.

^2^The CH scale was not used in this study.

CHQ-PF28 is a well-validated and reliable measure of HRQL in children from the age of 5 and normative data are available for the general U.S. population [28] and 4–13 years-olds from the Netherlands [29]. The Swedish version of the CHQ-PF50, which is a 50-item version of the CHQ, has been found to be valid and reliable in a group of Swedish children aged 9–16 years [30].

### Study-specific questionnaire

A study-specific questionnaire based on relevant literature on similar subjects [2,31] was devised by the authors. It included 21 items and covered the following topics: food items avoided, adverse food reactions, additional allergy/hypersensitivity besides food, and additional family members with food hypersensitivity. In order to get information about the prevalence of the allergic diseases asthma, eczema, and rhino conjunctivitis, the questions were formulated in a similar way as used in a multi-centred and international study on asthma and allergy, ISAAC [31]. The questionnaire can be obtained from the corresponding author on request. Prior to the data collection, a pilot test of the questionnaire was performed with nine parents, who were not included in the present study, and subsequently minor lexical adjustments were made. Some of the questions included were as follows:

"Is your child allergic or hypersensitive to any of the following?" (Possible answers: furred animal, pollen, dust/mite, food items, nickel, insects, drugs, detergents, other substances: Yes/No.)

"If your child is allergic or hypersensitive to any food items, what reactions or symptoms does she/he perceive? (Possible answers: not allergic or hypersensitive, eczema, rash, eye-nose-symptoms, itchy mouth, breathing difficulties, vomiting-diarrhoea-stomach ache, allergic shock, other)."

"Has anyone else in the child's family food allergy or food hypersensitivity? (Possible answers: Yes, one or both parents; Yes, one or more siblings; No, no one else in the family is food allergic/hypersensitive.)

The following four questions were used to evaluate the prevalence of the allergic diseases asthma, eczema and rhino conjunctivitis. The wordings of these questions were derived from the ISAAC study [31].

"Has your child had asthma, wheezing or whistling in the chest in the past 12 months? (Yes/No)"

"In the past 12 months, has your child had recurrent eczema or itchy rash for at least 6 months? (Yes/No)"

"In the past 12 months, has your child had a problem with sneezing, or a runny, or a blocked nose when she/he did not have a cold? (Yes/No)"

"In the past 12 months, has your child had a problem with itchy-watery eyes when she/he did not have a cold? (Yes/No)

In the present study, celiac disease is regarded as a food hypersensitivity condition and not as an allergic disease.

### Data analysis

The CHQ-PF28 and the quantitative data from the study-specific questionnaire were analysed using the SPSS 11.0 program. CHQ-PF28 data was processed using SPSS syntax with calculations provided by the principal developer of the CHQ, Jeanne Landgraf [28]. Internal consistency of the CHQ-PF28 health scales was tested by Cronbach's alpha and ranged in this study between 0.74 and 0.86 except for the scales *Mental Health (MH 0.68), General Health (GH 0.65) and Parental impact-Emotional (PE 0.65) *(*see *Table 1).

To test differences in proportions the Chi-square test was used. The Mann-Whitney U-test was used to assess differences in CHQ-PF28 means of rank scores between two independent groups. The strength of the relationships between CHQ-PF28 scores and allergy-associated parameters were calculated using Spearman rank correlation coefficients. A p-value <0.05 was considered to be statistically significant.

Stepwise multiple linear regression analyses were performed to examine the contribution of each allergy-associated parameter, age and gender, to the predictions of CHQ-PF28 scores. The regression analyses were carried out manually as follows. Single regression analyses were executed for each variable that showed a statistically significant difference in CHQ-PF28 means of rank scores using the Mann-Whitney U-test, or a statistically significant relationship with the CHQ-PF28 scores using the Spearman rank correlation coefficients. One variable at a time was entered into the model, starting with the variable showing the highest t- value. The procedure was repeated until the addition of another independent variable did not increase the explained variation (adjusted R-square), so the final models included the variables that enhanced the degree of explanation and were statistically significant.

The statistical analyses aimed to find and asses the relationships between variables from the study-specific questionnaire (i.e. number of offending food items, type and number of adverse food reactions, food reaction in the past 12 months, number of allergic diseases, number of additional allergies/hypersensitivities besides food, additional family members with food hypersensitivity, age and gender) and HRQL as indicated by six of the CHQ-PF28 scales (i.e. PhS = Physical summary, PsS = Psychosocial summary, PE = Parental impact-Emotional, PT = Parental impact-Time, FA = Family Activities and FC = Family Cohesion). These particular CHQ-PF28 scales were chosen as they summarize the child's HRQL (PhS, PsS) and show the impact of the child's health problems on parents (PE, PT) and family (FA, FC).

### Ethical considerations

The postal questionnaire that was distributed to the parents included an information letter about the purpose of the study and pointed out that participation was voluntary. The questionnaire was anonymous, i.e. no inquiries were made about personal data and the respondents could not be identified from the questionnaires.

In connection with a previous study [27] the parents agreed to be contacted again later on for further questions. The present study, as well as the previous one [27], was approved by the Director of School Administration in Tyresö municipality and ethical permissions for both studies were obtained from the research committee at Huddinge University Hospital (Dnr 122/03 and Dnr 404/1).

## Results

The results are based exclusively on information from parents of 134 schoolchildren with parent-reported food hypersensitivity. The children were 8–19 years of age (mean 12.5 years, SD 2.6). (Girls: 53%, n = 71, 8–18 years, mean 12.3 years, SD 2.3; Boys: 47%, n = 63, 8–19 years, mean age 12 years, SD 2.9.) The respondents were the food hypersensitive children's mothers (88%), fathers (7%) or both (5%).

### Allergy-associated parameters

The 134 children were reported to be hypersensitive to 1–6 food items each, with a median of two food items. The five most commonly reported food items were fruit/berries (34%), nuts (34%), peanut (33%), lactose (24%) and tomato (22%). The following offending food items were reported for 16-9% of the children: almond, egg, fish, carrot, soy, vegetables, gluten, milk and shellfish.

The children were reported to have 1–6 different types of adverse food reactions, with a median of two types of reactions per child. The most common type of food reaction reported was gastro-intestinal symptoms (50%), followed by OAS (Oral Allergy Syndrome, 47%). Of all 134 children, 45% (n = 60) had experienced at least one food reaction during the past 12 months (Table 2). The occurrence of food reactions during the past year differed according to what kind of reaction the child was reported to have, with the highest frequency (55%) among children with gastro-intestinal symptoms and the lowest frequency (36%) among those with food-induced breathing difficulties.

**Table 2:** Allergy-associated parameters among children with food hypersensitivity

	**N = 134**	**(%)**
**Food reactions**		
Gastro-intestinal symptoms	67	(50)
OAS – Oral Allergy Syndrome	63	(47)
Difficulty breathing	53	(40)
Skin symptoms	50	(37)
Anaphylaxis	20	(15)
Eye-nose symptoms	19	(14)
**Food reactions during the last 12 months**	**60**	**(45)**
**Allergic diseases**	**100**	**(75)**
Rhino conjunctivitis	69	(52)
Eczema	64	(48)
Asthma	44	(33)
**Additional allergy/hypersensitivity**	**95**	**(71)**
**Additional family member with food hypersensitivity**	**78**	**(58)**
Parent(s)	49	(37)
Sibling(s)	45	(34)

At least one of the allergic diseases asthma, eczema and rhino conjunctivitis was reported for 75% (n = 100) of the children (Table 2). Forty-six per cent (n = 61) of the children suffered from more than one of these diseases. According to what kind of food reaction the child experienced or was at risk of, the occurrence of allergic disease ranged between 70% (children with gastro-intestinal symptoms) and 87% (children with breathing difficulties).

No statistically significant gender differences were found in relation to the allergy-associated parameters, except for lactose. A higher prevalence of lactose intolerance was reported for girls (31%) than for boys (16%), p < 0.05.

### Health-related quality of life

Parents of food hypersensitive children with allergic diseases reported significantly lower HRQL in eight of the 14 CHQ-PF28 scales used in this study, compared with those with no allergic disease (Table 3). Significantly lower HRQL were seen on the child's physical dimension scales (PF = Physical Functioning, RP = Role-social limitations/Physical, BP = Bodily Pain, GH = General Health), the two scales showing parents' HRQL (PE = Parental impact-Emotional, PT = Parental impact-Time) and one scale showing family HRQL (FA = Family Activities). There were no significant differences between children with and those without allergic disease with regards to the scale scores representing the psychosocial dimension (REB = Role/social limitations-Emotional-Behavioural, BE = General Behaviour, MH = Mental Health, SE = Self Esteem) of the child's HRQL (Table 3).

**Table 3:** Parent-reported mean scores for the CHQ-PF28 in food hypersensitive schoolchildren with or without allergic disease(s)

**Scale**	**Total N = 134**		**Allergic disease ^**1 **^N = 100**		**No allergic disease N = 34**		***p*-value**
	**Mean**	**SD**	**Mean**	**SD**	**Mean**	**SD**	
Physical Functioning (PF)	87.6	20.2	83.6	21.9	99.3	3.8	.000
Role/social limitations – Emotional/Behavioural (REB)	90.2	20.0	89.8	19.8	91.1	20.6	NS
Role/social limitations – Physical (RP)	86.9	24.9	82.8	27.5	99.0	5.7	.000
Bodily Pain/discomfort (BP)	74.8	23.0	72.5	23.8	81.7	19.3	.048
Behaviour (BE)	68.1	17.0	67.5	17.6	70.1	15.4	NS
Mental Health (MH)	72.0	15.3	71.3	16.1	74.0	12.9	NS
Self Esteem (SE)	77.7	17.4	76.6	17.5	81.1	16.9	NS
General Health (GH)	70.9	23.4	66.3	23.5	84.	17.6	.000
Parental impact – Emotional (PE)	81.9	20.4	79.3	20.8	89.3	17.1	.003
Parental impact – Time (PT)	90.4	17.3	88.3	19.2	96.5	6.8	.029
Family Activities (FA)	85.5	19.2	83.8	19.4	90.4	17.9	.027
Family Cohesion (FC)	68.8	25.3	70.6	24.4	63.6	27.4	NS
Physical summary measure (PhS) ^2^	49.8	11.7	47.1	12.3	57.6	4.6	.000
Psychosocial summary measure (PsS) ^2^	49.6	9.4	49.2	9.6	50.5	8.8	NS

^1 ^Asthma, eczema, rhino conjunctivitis.

^2 ^The PhS and PsS measures are standardized so that all scores above and below 50 are above and below the average, respectively, of a general U.S. child population [28].

A gender comparison within the group of food hypersensitive children showed that the boys were reported to have significantly lower CHQ-PF28 scores than the girls in the following scales: Physical Functioning (83.9 SD 22.9 and 90.9 SD 16.9 respectively, p < 0.05), General Health (66.6 SD 24.0 and 74.7 SD 22.4 respectively, p < 0.05) and Physical Summary measure (47.2 SD 12.7 and 52.1 SD 10.4 respectively, p < 0.05). As regards the Mental Health scale the result was the opposite. Significantly lower scores were reported for the girls (68.6 SD 13.3 and 75.7 SD 16.6 respectively, p < 0.01).

### HRQL and the relationship with allergy-associated parameters, age and gender

Significant rank mean score differences in six CHQ-PF28 scales (PhS, PsS, PE, PT, FA and FC) in schoolchildren with or without reported allergy-associated parameters and gender differences are shown in Table 4. There were significantly lower PhS scores reported for children with food-induced difficulty breathing and male gender, indicating that these conditions involved the experience of physical limitations for the child. Table 5 demonstrates that the larger the number of offending food items, food reactions, allergic diseases, additional allergies/hypersensitivities besides food the lower the PhS scores, indicating decreased physical well-being.

**Table 4:** Significant rank mean score differences in six CHQ-PF28 scales^1 ^between schoolchildren with and without allergy-associated parameters^2^.

**Allergy-associated parameters**		***HRQL***^***3***^					
		
		***Child***		***Parent***		***Family***	
		**PhS**	**PsS**	**PE**	**PT**	**FA**	**FC**
Food reactions							
Gastro intestinal symptoms (N = 67)	Yes No *p*			77.6 86.1 0.033			
OAS – Oral Allergy Syndrome^4 ^(N = 63)	Yes No *p*		*51.6* *47.7* 0.016				
Difficulty breathing^4 ^(N = 53)	Yes No *p*	47.0 51.6 0.008	*52.6* *47.6* 0.006				*75.6* *64.3* 0.021
Anaphylaxis^4 ^(N = 20)	Yes No *p*		*55.3* *48.6* 0.003				
Additional family member(s) with food hypersensitivity (N = 78)	Yes No *p*			79.6 85.6 0.045	88.7 92.7 0.045	82.8 89.3 0.045	
Sibling(s) with food hypersensitivity (N = 45)	Yes No *p*		46.5 51.1 0.023		85.2 93.0 0.006		
Male gender (N = 63)	Yes No *p*	47.2 52.1 0.014					

^1 ^The six CHQ-PF28 health scales used in this study are: PhS = Physical Summary measure; PsS = Psychosocial Summary measure; PE = Parental impact-Emotional; PT = Parental impact-Time; FA = Family impact-Activities; FC = Family impact-Cohesion [28].

^2 ^Allergy-associated parameters tested not showing significant mean differences were: the food reactions skin symptoms and eye-nose symptoms; food reactions in the past 12 months; parent(s) with food hypersensitivity.

^3 ^HRQL = Health-related quality of life

^4 ^Score means shown in *italics *indicate higher score means, i.e. higher HRQL, when the allergy-associated parameter mentioned was present.

**Table 5:** Correlations between CHQ-PF28 scale scores and allergy-associated parameters. Spearman correlation coefficients with statistical significance between PhS, PsS, PE, PT, FA and FC^1 ^and number of offending food items, food-induced reactions, allergic diseases, additional allergies/hypersensitivities and years of age.

		***HRQL***					
		***Child***		**Parent**		**Family**	
		**PhS**	**PsS**	**PE**	**PT**	**FA**	**FC**
Offending food items (range 1–6)	rho *p*	-.397 0.000	*.225* 0.010			-.173 0.047	
Types of food reactions (range 1–6)	rho *p*	-.245 0.005	*.188* 0.031				*.190* 0.028
Allergic diseases (range 0–3)	rho *p*	-.500 0.000		-.319 0.000	-.280 0.001	-.354 0.000	
Additional allergies/hypersensitivity (range 0–8)	rho *p*	-.475 0.000		-.178 0.040	-.290 0.001	-.261 0.002	
Years of age (range 8–19)	rho *p*					*.189* 0.028	

^1 ^CHQ-PF28 health scales: PhS = Physical Summary score; PsS = Psychosocial Summary score; PE = Parental impact-Emotional; PT = Parental impact-Time; FA = Family impact-Activities; FC = Family impact-Cohesion [28].

^2 ^Positive correlation coefficients, shown in *italics*, indicate a positive relationship, i.e. the higher the number of offending food items/types of food reactions/years of age, the higher the HRQL scores.

To be the only child in the family with food hypersensitivity implied better psychosocial well-being (PsS) for the child than if she/he had sibling(s) with food hypersensitivity as well (Table 4).

There were significantly lower PE scores reported by the parents to children with gastro-intestinal symptoms, indicating emotional worry or concern as a result of the child's health problems (Table 4). The higher the number of allergic diseases and additional allergies/hypersensitivities besides food, the lower the scores on the PE and PT scales, i.e. the more worry and concern (PE) and limitation in time for personal needs (PT) were reported by the parents (Table 5).

There were significantly lower FA scores reported by the respondents who had additional family members with food hypersensitivity, indicating a negative impact on family activities (Table 4). Furthermore, the higher the number of offending food items, allergic diseases and additional allergies/hypersensitivities, the lower the FA scores and the younger the child the lower the FA scores, i.e. more disruption in family activities due to the child's health (Table 5).

### Allergy-associated parameters and HRQL enhancement

There were significantly higher scores on the PsS scale reported for the children who were reported to react to food with OAS, difficulty in breathing and/or anaphylaxis, indicating better psychosocial well-being for the children with these conditions (Tables 4). Moreover, the higher the number of offending food items and different types of food reactions the higher the PsS scores, indicating better psychosocial well-being for children with complex food hypersensitivity (Table 5).

Finally, there were significantly higher FC scores (families' ability to get along) reported by parents of children with food-induced breathing difficulties compared with the remaining children (Table 4). The higher the number of types of food reactions, the better the families ability to get along (Table 5).

### Predictors of HRQL outcome

In order to find factors that predict impact on HRQL, stepwise multiple linear regression analyses were carried out. The six CHQ-PF28 scales were used as dependent variables. The parameters in Tables 4 and 5 shown to be statistically significant were used as independent variables.

Three variables explained 30.8% of the variance of the PhS scale: number of allergic diseases, number of additional allergies/hypersensitivities and male gender (Table 6). Thus, the higher the number of allergy-associated conditions in addition to food hypersensitivity, the lower the physical HRQL. Also, male gender predicted lower physical HRQL than female gender. Regarding the PsS scale, 9% of the variance was explained by two variables: if the child had food-induced breathing difficulties it predicted higher psychosocial HRQL, while having sibling(s) with food hypersensitivity predicted lower psychosocial HRQL for the child of interest (Table 6).

**Table 6:** Prediction of HRQL outcome by means of six CHQ-PF28 scales. Stepwise multiple linear regression analyses by means of the CHQ-PF28 scales PhS, PsS, PE, PT, FA and FC^1^.

	**B Coeff**.	**(95% CI)**	**t**	***p*-value**	**R Square**	**Adj. R Square**
**Dependent variable: Child Physical Summary (PhS)**						
Independent variables:						
Number of allergic diseases (0–3)	-3.262	(-5.480 – -1.044)	-2.910	0.004		
Number of additional allergies/hypersensitivities (0–8)	-2.327	(-3.692 – -0.962)	-3.373	0.001		
Male gender (0 = no, 1 = yes)	-4.633	(-8.054 – -1.212)	-2.680	0.008		
Model summary					0.324	0.308
**Dependent variable: Child Psychosocial Summary (PsS)**						
Independent variable:						
Food-induced breathing difficulties^2 ^(0 = no, 1 = yes)	*4.501*	(1.297 – 7.705)	2.779	0.006		
Sibling(s) with food hypersensitivity (0 = no, 1 = yes)	-3.994	(-7.311 – -0.677)	-2.382	0.019		
Model summary					0.106	0.093
**Dependent variable: Parental impact – Emotional (PE)**						
Independent variables:						
Number of allergic diseases (0–3)	-6.957	(-10.264 – -3.651)	-4.162	0.000		
Food-induced gastro-intestinal symptoms (0 = no, 1 = yes)	-9.932	(-16.426 – -3.438)	-3.026	0.003		
Model summary					0.156	0.143
**Dependent variable: Parental impact – Time (PT)**						
Independent variables:						
Number of allergic diseases (0–3)	-5.852	(-8.657 – -3.046)	-4.127	0.000		
Sibling(s) with food hypersensitivity (0 = no, 1 = yes)	-8.488	(-14.321 – -2.656)	-2.879	0.005		
Model summary					0.156	0.143
**Dependent variable: Family impact – Activities (FA)**						
Independent variables:						
Number of allergic diseases (0–3)	-6.465	(-9.616 – -3.315)	-4.060	0.000		
Additional family member(s) with food hypersensitivity (0 = no, 1 = yes)	-6.653	(-12.934 – -0.371)	-2.095	0.038		
Model summary					0.137	0.124
**Dependent variable: Family impact – Cohesion (FC)**						
Independent variable:						
Food-induced breathing difficulties^2 ^(0 = no, 1 = yes)	*11.363*	(2.661 – 20.064)	2.583	0.011		
Model summary					0.048	0.041

^1^CHQ-PF28 health scales: PhS = Physical Summary score; PsS = Psychosocial Summary score; PE = Parental impact-Emotional; PT = Parental impact-Time; FA = Family impact-Activities; FC = Family impact-Cohesion [28].

^2^Positive beta coefficients, shown in *italics*, indicate higher score means, i.e. higher HRQL, when the allergy-associated parameter food-induced breathing difficulties was present.

Fourteen per cent of the variances of the two scales concerning impact on parents' HRQL (PE = emotional impact and PT = time impact) were explained by two variables each. A high number of allergic diseases predicted low HRQL in both these scales. Moreover, the variable food-induced gastro-intestinal symptoms predicted low HRQL in PE and the variable sibling(s) with food hypersensitivity predicted low HRQL in PT (Table 6).

Twelve per cent of the variance of the FA scale was explained by two variables: number of allergic diseases and additional familymember(s) with food hypersensitivity – both predicting lower family HRQL, i.e. more disruption in family activities due to the child's health. Finally, 4% of the variance of the FC scale was explained by the variable food-induced breathing difficulties, i.e. this kind of food reaction in the child predicted higher family HRQL concerning the family's ability to get along (Table 6).

## Discussion

This paper focuses on parent-reported health-related quality of life (HRQL) in families with a food hypersensitive schoolchild and the relationships between allergy-associated parameters and HRQL scores.

The children in the present study were hypersensitive to 1–6 defined food items and the most common food reaction was gastro-intestinal symptoms. The majority (75%) of the children also suffered from allergic disease(s). The presence of allergic disease was found to be an important predictor of low child, parental and family HRQL, i.e. they were reported to have lower HRQL scores on several of the CHQ-PF28 scales compared with children with no such disease in addition to their food hypersensitivity.

### Children's physical and psychosocial HRQL

The group of children suffering from allergic disease in addition to food hypersensitivity was reported to have lower physical HRQL compared with those without such disease. Also Sicherer et al have shown that atopic disease additional to food allergy has an impact on the General Health scale [25]. This is in concordance with our previous study [2] showing lower HRQL on the SF-36 scales Bodily Pain and General Health for the food hypersensitive children with additional allergic disease. This is not surprising as the allergic diseases asthma, eczema and rhino conjunctivitis to a great extent are physical disorders with somatic symptoms.

Looking at the food hypersensitive group without allergic diseases, the physical HRQL scores (CHQ-PF28) in this study did not seem low compared with a general U.S. population of children 5–18 years of age [28] and a general Netherlands population of children 4–13 years of age [29]. Thus, the present study does not suggest any physical HRQL deterioration among food hypersensitive children as long as there is no additional allergic disease.

The psychosocial HRQL scores in the present study showed no significant differences between those with and those without an allergic disease in addition to their food hypersensitivity. In comparison with the U.S. [25] and the Netherlands [29] general populations mentioned above, three of the four psychosocial HRQL scales for the child (REB, BE and MH) seem to show lower scores for the food hypersensitive children, suggesting psychosocial HRQL deterioration for food hypersensitive children whether additional allergic disease is present or not. This is in concordance with the results from our previous study [2], showing lower psychosocial HRQL (SF-36) for the hypersensitive adolescents compared with "healthy" adolescents, regardless of additional allergic disease. From this one may draw the conclusion that food hypersensitivity by itself is associated with psychosocial HRQL deterioration despite the consequences of additional allergic disease.

Avery et al [24] have shown that food allergic children can experience fear of adverse events and anxiety about eating. In the present study more than half (55%) of the children had not experienced any food reactions in the past 12 months. Still, all the children were reported to have lower psychosocial HRQL than the general population samples mentioned [25,29]. This may suggest that it is rather the risk of food reactions and measures to avoid them that is associated with lower HRQL than the clinical reactivity induced by food intake.

It is not unusual that children's hypersensitivity will get less severe over the years and that they even can grow out of it, as they grow older. This might explain the association found between age and HRQL, i.e. the lower the age the lower the scores on the FA (Family Activities) scale. However, in the regression analysis age did not turn out to be significantly associated with any HRQL scale.

Male gender implied significantly lower physical HRQL compared with female gender but female gender involved lower scores on the Mental Health scale. This finding differ partly from the results of our previous study [2], in which the food hypersensitive adolescent females to a greater extent reported lower HRQL in both the physical and the mental SF-36 scales. This inconsistency may be due to dissimilar responding groups (parents and adolescents, respectively). It is known that parental and child reports on HRQL can differ [32-34]. For example, parents may have limited knowledge concerning their children's HRQL, especially their psychosocial well-being [32]. As Williams [35] suggests, girls incorporate a chronic condition with their social identity and boys tend to diminish the importance of the condition to avoid stigmatisation. Alternatively, parents may overlook more physical limitations in girls than in boys. Anyhow, these findings require further exploration.

### Parental and family HRQL

Bollinger et al [36] have shown that food allergy has a significant impact on daily activities of food allergic children and their families. Moreover, Sicherer et al [25], who also used the CHQ-questionnaire, have shown that parents of food allergic children experience worry and concern (PE) and limitations in family activities (FA) due to their child's health problem. None of these studies, however, found that additional allergic disease had any significant impact on parents and families. Yet, the results from the present study indicate that additional allergic disease do have a negative impact on parental and family HRQL, i.e. on worry and concern (PE), limitation in time for personal needs (PT), and disruption in family activities (FA). When comparing our results with those of Sicherer et al different conclusions were drawn on whether additional allergic disease impairs the parents' and families' HRQL. This difference might be explained by the fact that Sicherer et al [25] in addition to their significance level of p < 0.05 also used the criterion of a 10-point difference in CHQ scores for statistical significance. In the present study, the PE-scale showed a 10-point difference and therefore fulfils also this criterion. The HRQL scores in both studies show a similar pattern and it seems likely that caring for a food hypersensitive child with somatic health problems may involve both emotional and time-consuming efforts. Still, the divergent results emphasise the need for further investigations on the significance of allergic diseases for families with food hypersensitive children.

Studies have shown that in families with a food hypersensitive child, it is common that more than one family member suffers from adverse food reactions [27,37]. In the present study, 58% of the families had more than one family member with food hypersensitivity and the regression analyses showed that having more than one family member with food hypersensitivity involved increased family strain, i.e. negative impact on the child's psychosocial HRQL (PsS), parents' time for personal needs (PT) and family activities (FA). The research on risks for the sibling of a chronically ill child is comprehensive [38-41], but the linkage between psychosocial HRQL deterioration and being more than one child in the family with a chronic disorder has, to our knowledge, not previously been reported. Although further research is needed to verify the coherence in these findings, it should be noticed that having more than one family member with food hypersensitivity is associated with HRQL deterioration.

### Gastro-intestinal symptoms, breathing difficulties and HRQL

From the statistical regression analyses it can be concluded that there were two kinds of food reaction with significant impact on HRQL, i.e. gastro-intestinal symptoms and breathing difficulties. Gastro-intestinal symptoms were significantly associated with parents' worry and concern. Such hypersensitivity reactions are often diffuse and enduring and the causal connection can be hard to confirm. Moreover, there is almost no medication existing for these kinds of symptoms. Consequently, parents may find it difficult to reduce the risk of inconvenience and to mitigate the child's pain, and this may account for the association between a child's gastro-intestinal symptoms and deterioration of parent's emotional HRQL.

In contrast to gastro-intestinal symptoms, the presence of food-induced breathing difficulties seems to improve two aspects of HRQL, i.e. the psychosocial well-being for the child (PsS) and family cohesion (FC). These somewhat puzzling results are partly in line with the findings of Rydström et al [42], who showed that Swedish children with asthma reported less impairment of quality of life in the domain of emotions than in activities. Concerning families' ability to get along, Case-Smith [43] has shown that although a child with a chronic medical condition is a strain to the whole family, this may strengthen the family cohesion. Also Sicherer et al [25] showed that family cohesion was stronger in families with peanut allergic children compared with a population sample. According to Reichenberg and Broberg [44], in families with asthmatic children the parent's ratings of family cohesion is clearly related to their child's psychological adjustment, i.e. high family cohesion is related to high scores on global self-worth for the child. In contrast to the gastro-intestinal symptoms, food induced breathing difficulties can be life threatening, the triggers are usually simple to identify and there are medical resources to treat the symptoms. Even though the risk of an acute reaction might cause worry and fear one knows what to do when it occurs. It may be speculated that individuals and families with this kind of hypersensitivity problem develop coping strategies built on cooperation and communication with those around and between family members. Also LeBovidge et al [45], who assessed parental response to children's food allergies, have highlighted the significance of parents' agreement and support from spouses and extended families. Such a hypothesis should be tested, and may generate valuable knowledge of how best to support families with food hypersensitive children.

### Methodological considerations

In the area of social and behavioural sciences, the possibility to predict the variance in the dependent variable is generally low, and an explained variance of 25% is considered high [46]. In the present study, accuracy of prediction as determined by adjusted R-square values was between 30.8% and 4.1%. There are of course predictors of importance for HRQL in food hypersensitivity missing in our regression models. Two questionnaires for measuring the burden in families with a child with food allergy have recently been developed [45,47]. These questionnaires, not available when the present study was conducted, point out some issues of importance for the food hypersensitive family, such as parental frustration over others' lack of appreciation for the seriousness of the hypersensitivity condition and parental coping. By addressing such issues in a future study perhaps the percentage of the variance explained would increase. Furthermore, although statistical significances are strong, some of the results show small score differences between groups and one can discuss the question as to at what level the differences are of importance. It is also important to discuss the clinical relevance of the results and look into this issue more closely in future studies.

The choice to use the short version of the CHQ (i.e. CHQ-PF28) instead of the longer CHQ-PF50 may be a subject of debate. According to Landgraf [28], CHQ-PF28 was derived from CHQ-PF50 to be used in larger population-based studies (N > 60). However, a Netherlands study [29] has shown that the summary measures in CHQ-PF28 and in CHQ-PF50 were comparable, but not each separated CHQ scale. A shorter questionnaire is preferable for many reasons: thus we chose to use the CHQ-PF28 and in the regression analyses the two summary measures for the children's quality of life were used, together with the scales that specifically measure the parents' and the families' HRQL. Subsequently, the results from the parents' and families' HRQL scales should be interpreted with some caution as these scales are not yet fully validated.

The scales MH, GH and PE showed Cronbach's alpha <0.70 but as they still show medium internal consistency (0.65 – 0.68) they were included in the analyses. According to Nunnally and Bernstein [48] comparisons between groups can be performed with lower internal consistency (0.50–0.70) than in comparisons between individual scores. Furthermore, two of the scales (MH and GH) are included in the summary measures PsS and PhS, which are used in the main part of the analyses.

## Conclusion

The results from this study are based on parent-reported HRQL in families with food hypersensitive schoolchildren. The variance in the child's physical HRQL was to a considerable extent explained by the presence of allergic disease. However, food hypersensitivity by itself was associated with deterioration of child's psychosocial HRQL, regardless of additional allergic disease. The results suggest that it is rather the risk of food reactions and measures to avoid them that are associated with lower HRQL than the clinical reactivity induced by food intake. Therefore, food hypersensitivity must be considered to have a strong psychosocial impact.

## Competing interests

The authors declare that they have no competing interests.

## Authors' contributions

BM and GN conceived of the study. All authors made substantial contributions to conception, planning and design. BM carried out the acquisition, analysis and interpretation of data. BM drafted the manuscript. GN and SA have been involved in revising it critically for important intellectual content. All authors read and approved the final manuscript.
